# Proton-dependent inhibition of the cardiac sodium channel Na_v_1.5 by ranolazine

**DOI:** 10.3389/fphar.2013.00078

**Published:** 2013-06-21

**Authors:** S. Sokolov, C. H. Peters, S. Rajamani, P. C. Ruben

**Affiliations:** ^1^Molecular Cardiac Physiology Group, Department of Biomedical Physiology and Kinesiology, Simon Fraser UniversityBurnaby, BC, Canada; ^2^Department of Biology, Cardiovascular Therapeutic Area, Gilead Sciences, Inc.Fremont, CA, USA

**Keywords:** ranolazine, acidosis, cardiac sodium channel, Na_v_1.5, electrophysiology

## Abstract

Ranolazine is clinically approved for treatment of angina pectoris and is a potential candidate for antiarrhythmic, antiepileptic, and analgesic applications. These therapeutic effects of ranolazine hinge on its ability to inhibit persistent or late Na^+^ currents in a variety of voltage-gated sodium channels. Extracellular acidosis, typical of ischemic events, may alter the efficiency of drug/channel interactions. In this study, we examined pH modulation of ranolazine's interaction with the cardiac sodium channel, Na_v_1.5. We performed whole-cell path clamp experiments at extracellular pH 7.4 and 6.0 on Na_v_1.5 transiently expressed in HEK293 cell line. Consistent with previous studies, we found that ranolazine induced a stable conformational state in the cardiac sodium channel with onset/recovery kinetics and voltage-dependence resembling intrinsic slow inactivation. This interaction diminished the availability of the channels in a voltage- and use-dependent manner. Low extracellular pH impaired inactivation states leading to an increase in late Na^+^ currents. Ranolazine interaction with the channel was also slowed 4–5 fold. However, ranolazine restored the voltage-dependent steady-state availability profile, thereby reducing window/persistent currents at pH 6.0 in a manner comparable to pH 7.4. These results suggest that ranolazine is effective at therapeutically relevant concentrations (10 μM), in acidic extracellular pH, where it compensates for impaired native slow inactivation.

## Introduction

Ranolazine is a piperazine derivative that was clinically approved by the FDA in 2006 for treatment of angina pectoris. It decreases late sodium currents (I_Na_) through cardiac sodium channel, Na_v_1.5, and thereby reduces calcium influx through the sodium-calcium exchanger NCX during reverse mode activity (Sossalla et al., 2008). Recently, more potential applications for ranolazine have been explored and reported. Ranolazine demonstrated antiarrhythmic properties in both atria and ventricles (Wu et al., 2004; Undrovinas et al., 2006; Burashnikov et al., 2007; Dobrev and Nattel, 2010). The proposed basic mechanism underlying ranolazine's antiarrythmic action in ventricles is inhibition of late I_Na_ (Wasserstrom et al., 2009; Undrovinas et al., 2010). In the atria, use-dependent inhibition of peak I_Na_ as well as I_Kr_ is thought to play an important role in treatment of atrial fibrillation, in addition to suppression of late I_Na_ (Burashnikov et al., 2007; Sossalla et al., 2010). Additionally, ranolazine displayed cardioprotection during ischemia (Hale et al., 2006, 2008; Stone et al., 2010), and effectively diminished late I_Na_ in Long QT syndrome type 3 mutations (Fredj et al., 2006; Moss et al., 2008; Kahlig et al., 2010; Huang et al., 2011).

Ranolazine may also be effective in non-cardiac tissue. It blocks the skeletal muscle sodium channel, Na_v_1.4, (Wang et al., 2008) and shows increased potency for paramyotonia congenita mutants (El-Bizri et al., 2011). Ranolazine inhibits neuronal channels, Na_v_1.7 (Rajamani et al., 2008; Wang et al., 2008) and Na_v_1.1, (Kahlig et al., 2010) and shows potent reduction of persistent currents in GEFS^+^, SMEI, and FHM3 mutants (Kahlig et al., 2010). Ranolazine also decreases cell excitability of dorsal root ganglion neurons (Estacion et al., 2010; Hirakawa et al., 2012), thus demonstrating an analgesic utility in the treatment of neuropathic pain (Gould et al., 2009). We previously reported the effects of ranolazine on the brain isoform, Na_v_1.2 at normal and acidic extracellular pH (Peters et al., 2013).

Similar to local anesthetic drugs, ranolazine's action on voltage-gated ion channels (including some Ca^2+^ and K^+^ channels, see Antzelevitch et al., 2011, for review) involves use-dependent as well as tonic block. However, therapeutic benefits of ranolazine for cardioprotection in ischemic conditions are attributed to its distinctive ability to diminish late sodium current in a variety of channels (Belardinelli et al., 2006). Ranolazine's IC_50_ for peak I_Na_ in Na_v_1.5 is reported to be several hundred μM, while late Na^+^ current is inhibited in the 7–10 μM range (Rajamani et al., 2009; Antzelevitch et al., 2011), close to therapeutically achieved plasma concentrations (2 ÷ 6 μM, Chaitman, 2006; 5.8 μM, Antzelevitch et al., 2011). Thus, while not exhibiting prominent isoform specificity, ranolazine possesses an intriguing type of functional specificity.

In the present study we examined effects of ranolazine on cardiac sodium channel Na_v_1.5 transiently expressed in HEK293 cells as well as the effects of extracellular acidification to pH 6.0, typically exhibited during myocardial ischemia (Maruki et al., 1993), on drug channel interaction. We suggest the capacity of ranolazine to inhibit late I_Na_ at therapeutically relevant concentrations (10 μM) may be related to its ability to potentiate slow inactivation. Ranolazine shifts the midpoint of the steady-state availability curve to more hyperpolarized voltages, accelerates entry into, and slows recovery from, a conformational state that shares kinetic- and voltage-dependent profiles of Na_v_ channel slow inactivation. Extracellular acidification impairs inactivation states of Na_v_1.5, including slow inactivation, leading to an increase in persistent current. Ranolazine remedies this deficiency, restoring inactivation over a time course of seconds to tens of seconds.

## Methods

### Expression of hNa_v_1.5 in HEK293 cells

The human variant of the cardiac sodium channel, hNa_v_1.5, was in the pRC-CMV vector (A.L. George, Vanderbilt University, Nashville, TN). Rat β_1_ subunit was in the pBK/CMV vector. HEK293 cells were cultured using media comprising DMEM (Gibco), FBS 20% (Gibco), and 10,000 U penicillin/streptomycin solution (Sigma). Cells were transiently transfected (Polyfect, Qiagen) with the hNa_v_1.5 α-subunit, β_1_ subunit, and enhanced Green Fluorescent Protein (pEGFP, graciously provided by Brett Adams, Utah State University, Logan, UT) to identify channel expression. Fluorescing cells were used for recording 24–36 h after transfection.

### Materials

Ranolazine was obtained from Gilead Sciences (Foster City, CA) in powder form, diluted to 100 mM stock in 0.1 M HCl, aliquoted at 10 mM and stored at −20°C. Working concentrations of 10 or 100 μM were freshly prepared in bath solution. pH was readjusted before performing electrophysiological experiments.

### Electrophysiology

Ionic currents were measured with whole-cell patch clamp using an EPC-9 amplifier, an ITC-16 interface, and an iMac running Patchmaster (HEKA, Lambrecht, Germany). Data were sampled at 50 kHz and low pass filtered at 10 kHz. Pipettes were made from borosilicate glass (Sutter Instruments, Novato, CA), coated with dental wax, and fire polished to a resistance of 1–1.5 MΩ. Series resistance, Rs, was typically 3 MΩ or less, Rs greater than 3 MΩ was compensated by 60–75%. Only cells with a seal resistance of 1 GΩ or greater were used. All measurements were conducted at room temperature (22°C).

The pipette solution contained (in mM): 130 CsF, 10 NaCl, 10 EGTA, and 10 HEPES adjusted to pH 7.4 with CsOH. The extracellular saline contained (in mM): 140 NaCl, 4 KCl, 2 CaCl2, 1 MgCl2, and 10 HEPES (pH 7.4) or MES (pH 6.0). pH was adjusted with CsOH.

A −130 mV holding potential was used for most voltage protocols except 10 and 25 Hz use-dependent trains, where an intermittent −100 mV resting potential was used between test pulses. We recognize −130 mV is more negative than the physiological resting potential in cardiac myocytes of −80 mV and the pathological resting potential of −65 mV during ischemia and we thus may miss some physiologically relevant information. However, the nature of our measurements require the channels to be in a “ground” state in which they are all closed and fully recovered from inactivation. All test pulses were to −10 mV. Cells were perfused at the holding potential for 5 min after whole-cell configuration was achieved before recordings started to allow dialysis of internal solution and stabilization of current amplitude. In matched pair experiments (Figure 7: ramps; Figure 9: tonic block and steady-state slow inactivation) control data were obtained first, followed by perfusion of cells for 5 min with either 10 or 100 μM ranolazine while holding at −130 mV. Voltage protocols were then repeated in the presence of ranolazine. This procedure minimized run down and gradual deterioration of the seal during prolonged recordings. A −P/4 leak subtraction was used for most protocols except use-dependent trains, ramps, and slow inactivation kinetics protocols. Details of voltage pulse protocols are given in the figure legends. Fit parameters are reported in Tables A1–A9 (Appendix).

Student's *t*-tests with two-tailed *p*-values were used for statistical analysis using the Instat software package (GraphPad Software, San Diego, CA). Results are presented as means ± SEM unless otherwise stated. Statistical significance is assumed to be *p* < 0.05 if not specifically given.

## Results

### Activation and fast inactivation

**Figure 1:**
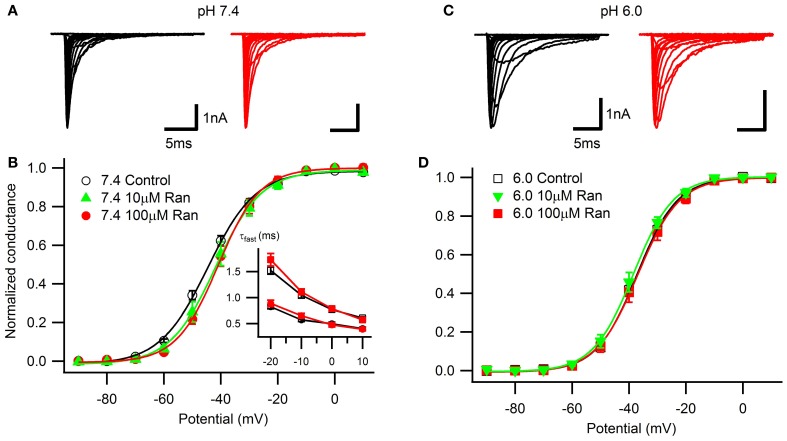
**Voltage-dependence of activation and open-state inactivation time constants**. Representative currents in response to 20 ms depolarizations in 10 mV steps from −130 mV at extracellular pH 7.4 **(A)** and 6.0 **(C)** in drug-free conditions (black) and with 100 μM ranolazine (red). **(B)** Conductance-voltage relationships at pH 7.4 in control (open black circles), 10 μM ranolazine (solid green triangles), and 100 μM ranolazine (solid red circles). **(D)** Conductance-voltage relationships at pH 6.0 in control (open black squares), 10 μM ranolazine (solid green triangles), and 100 μM ranolazine (solid red squares). Fit parameters are summarized in Table A1. (**B**, inset) Time constants of open-state inactivation were obtained by fitting current decay with a single exponential function in control (open black symbols) and with 100 μM ranolazine (solid red symbols) at pH 7.4 (circles) and 6.0 (squares). Time constants are reported in Table A2.

We examined the effects of extracellularly applied ranolazine on Na_v_1.5, transiently co-expressed in HEK293 cells along with the β_1_ subunit and eGFP marker. We studied ranolazine action in two different extracellular pH conditions: pH 7.4 (Figures 1A,B) corresponding to normal physiological conditions and pH 6.0 (Figures 1C,D) that approximates extracellular pH during ischemic cardiac events.

Consistent with our previous studies (Jones et al., 2011; Vilin et al., 2012) and others (Murphy et al., 2011), we observed a ~7 mV depolarizing shift in the voltage-dependence of activation at pH 6.0 (Figure 1; Table A1). At pH 7.4, the presence of 10 or 100 μM ranolazine in the extracellular solution caused a small but statistically significant depolarizing shift of the activation curves. Ranolazine did not significantly affect the V_1/2_ of activation at pH 6.0 compared to Ranolazine at pH 7.4.

Open-state fast inactivation kinetics were quantified by fitting current decay with an exponential function from test pulses between −20 and +10 mV (Figure 1B, inset; Table A2). Acidic pH significantly slowed channel fast inactivation. 100 μM ranolazine did not significantly affect fast inactivation time constants at either pH.

**Figure 2:**
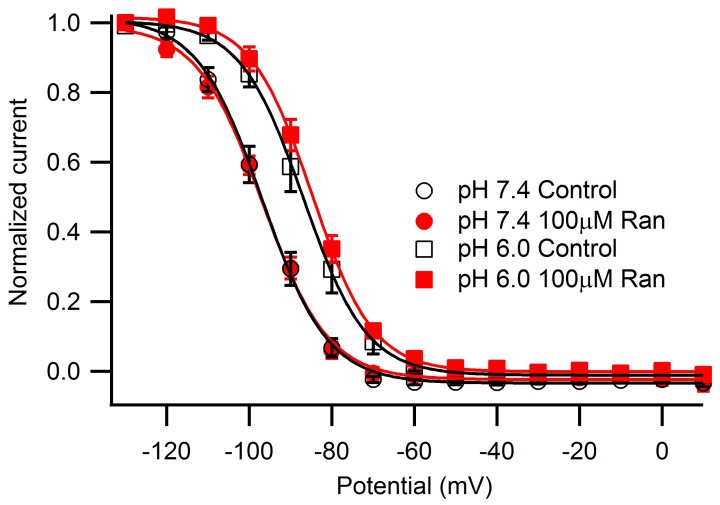
**Steady-state fast inactivation induced by 500 ms conditioning pulses ranging from −130 mV to +10 mV in 10 mV intervals and measured by a test pulse to −10 mV**. Channels were held at −130 mV for 10 s between sweeps. pH 7.4 control (open black circles) differed significantly from pH 6.0 control (open black squares). 100 μM ranolazine had no effect on steady-state fast inactivation (pH 7.4, solid red circles; pH 6.0, solid red squares). Fit parameters are summarized in Table A3.

Steady-state fast inactivation was assessed after 500 ms conditioning pulses to voltages from −130 to +10 mV (Figure 2). Acidic pH caused a ~10 mV depolarizing shift of the V_1/2_ with no effect on the apparent valence (Table A3). 100 μM ranolazine did not significantly shift the V_1/2_ or apparent valence at either pH.

**Figure 3:**
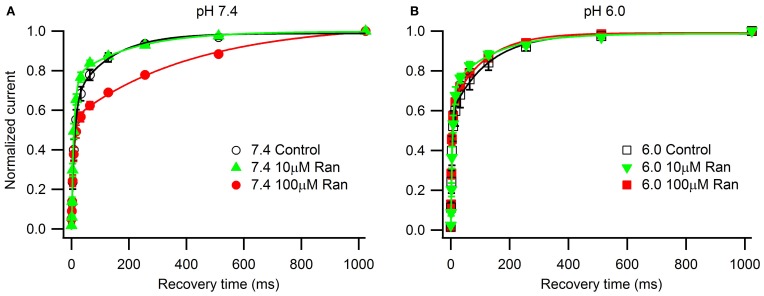
**Fast inactivation recovery at −130 mV**. Fast inactivation was induced by a 500 ms conditioning pulse to −10 mV. Cells were then hyperpolarized to −130 mV for 0–1000 ms and current measured with a −10 mV test pulse was plotted as a function of recovery pulse duration and fitted with a double exponential function. **(A)** pH 7.4 in control (open black circles), 10 μM ranolazine (solid green triangles), and 100 μM ranolazine (solid red circles). **(B)** pH 6.0 in control (open black squares), 10 μM ranolazine (solid green triangles), and 100 μM ranolazine (solid red squares). Fit parameters are in Table A4.

We next studied the kinetics of fast inactivation recovery after a 500 ms conditioning pulse to −10 mV measured by 20 ms test pulses to −10 mV applied at various intervals (Figure 3). Consistent with previous studies (Jones et al., 2011; Vilin et al., 2012), we detected accelerated fast inactivation recovery kinetics at pH 6.0 (Table A4). Interestingly, 100 μM ranolazine induced a profound slow component of recovery at pH 7.4, but not at pH 6.0 (Figures 3A,B).

### Use-dependent inhibition by ranolazine

**Figure 4:**
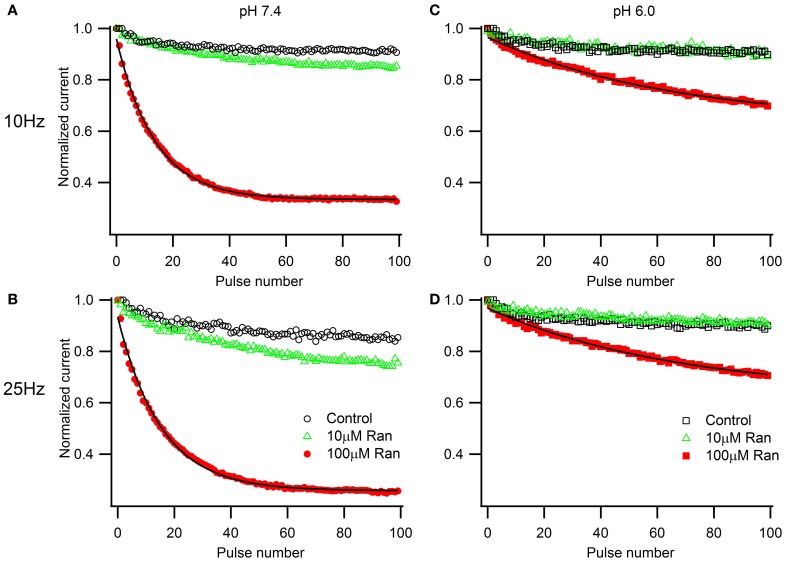
**Use-dependent inactivation induced by one hundred 5 ms pulses to −10 mV applied at 10 Hz (A,C) or 25 Hz (B,D) from holding potential −100 mV at extracellular pH 7.4 (A,B) and 6.0 (C,D)**. Error bars removed for clarity. Use-dependence in presence of 100 μM ranolazine (red symbols) was fit with single exponential function (solid lines). Fit parameters are reported in Table A5.

We next examined the extracellular pH effects on use-dependent channel inactivation and block by ranolazine. At first, we used high frequency depolarization trains (10 and 25 Hz, Figure 4) that many studies employ to assess use-dependent block by ranolazine, local anesthetics, and other compounds with state-dependent action (Fredj et al., 2006; Kahlig et al., 2010; El-Bizri et al., 2011; Huang et al., 2011; Hirakawa et al., 2012).

In the presence of 100 μM ranolazine at pH 7.4, a series of 100 pulses produced 67 ± 1% peak current inhibition at 10 Hz and 74 ± 3% at 25 Hz (Table A5). At pH 6.0 the degree of use-dependent inhibition by ranolazine was significantly reduced at both pulsing frequencies. This result correlates with our observation of drug-induced slow recovery component at pH 7.4, but not at pH 6.0 (Figure 3). Use-dependent inhibition developed 4–5 fold slower at pH 6.0 than at pH 7.4, and subsequently did not reach steady-state equilibrium within 100 pulses.

**Figure 5:**
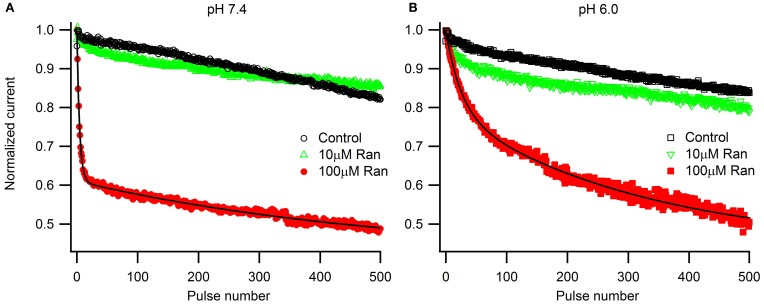
**Use-dependent inactivation by trains of five hundred 300 ms pulses to −10 mV applied at 1 Hz from holding potential −130 mV at extracellular pH 7.4 (A) and 6.0 (B)**. Error bars removed for clarity. Data in 100 μM ranolazine (red symbols) was fit with double exponential functions (solid lines). Fit parameters are summarized in Table A6.

To assess the pH-dependence of ranolazine effect at more physiologically relevant conditions, we applied trains of 500 pulses 300 ms in length at 1 Hz, roughly corresponding to cardiac action potential frequency and duration (Figure 5). pH 6.0 significantly slowed inhibition kinetics by 100 μM ranolazine (Figure 5, Table A6). Interestingly, use-dependent block by 100 μM ranolazine reached similar magnitudes at pH 7.4 and 6.0 by the 500th pulse (pH 7.4: 51 ± 4%; pH 6.0: 50 ± 4%).

### Ranolazine effects on late sodium current and window current

**Figure 6:**
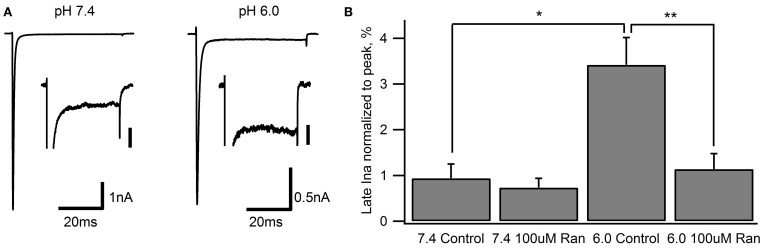
**Late Na^+^ current during 50 ms depolarization to −10 mV from a −130 mV holding potential**. Representative traces from recordings at extracellular pH 7.4 and 6.0 are shown in **(A)** Inset scale bars are 40 pA. **(B)** Late I_Na_ measured at 45–50 ms normalized to peak current in drug-free conditions (pH 7.4: 0.94 ± 0.34%, *n* = 7; pH 6.0: 3.4 ± 0.6%, *n* = 7) and with 100 μM ranolazine (pH 7.4: 0.73 ± 0.17%, *n* = 10; pH 6.0: 1.1 ± 0.4%, *n* = 8). Statistically significant differences are indicated with asterisks.

The clinical benefits of ranolazine are predominantly ascribed to its ability to block late or window Na^+^ currents (Reddy et al., 2010; Antzelevitch et al., 2011). We measured late sodium current (late I_Na_) with 50 ms depolarizations to −10 mV from a holding potential of −130 mV (Figure 6). Fifty sweeps were averaged to improve the signal to noise ratio. In line with previous studies (Magyar et al., 2004; Huang et al., 2011) we found that Na_v_1.5 channels exhibit a very small late I_Na_ at pH 7.4 (0.94 ± 0.34% of peak current amplitude). In the presence of 100 μM ranolazine, we observed a small reduction of late I_Na_ (0.73 ± 0.17%). This effect was, however, not statistically significant (*p* > 0.05).

Extracellular pH 6.0 substantially reduces Na_v_ channel peak current amplitude (Jones et al., 2011; Murphy et al., 2011; Vilin et al., 2012). When normalized to peak current, late I_Na_ was significantly increased to 3.4 ± 0.6% at pH 6.0 (Figure 6). Addition of 100 μM ranolazine to the extracellular solution significantly reduced the relative late I_Na_ to 1.1 ± 0.4% (*p* < 0.01).

**Figure 7:**
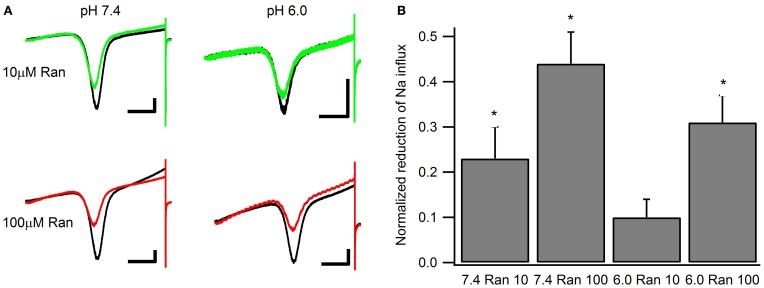
**Window currents measured with 500 ms ramps (0.3 mV/ms) from −130 to +20 mV. (A)** Representative matched pairs before and after perfusion of either 10 μM ranolazine (top) or 100 μM ranolazine (bottom). Scale bars are 100 ms and 50 pA. **(B)** Total charge flow was calculated by integrating current traces after subtracting the linear leak component. Charge for every cell was then normalized to that in the absence of ranolazine and plotted as a function of drug and pH. Statistically significant reductions in charge influx are indicated with asterisks.

Window currents arise due to partial activation and incomplete inactivation of voltage-gated ion channels at intermediate membrane potentials. To examine the effects of ranolazine on window currents, we applied 0.3 mV/ms ramps before and after perfusion of 100 μM of the drug (Figure 7). Fifty sweeps were averaged to improve the signal to noise ratio (Figure 7A). In these matched recordings, 100 μM ranolazine significantly reduced inward sodium influx (pH 7.4: 44 ± 7%; pH 6.0: 31 ± 6%). The difference between reduction of window currents at pH 7.4 and 6.0 was not statistically significant. The effect of 10 μM ranolazine was significant at pH 7.4 (23 ± 3%, *p* = 0.02), but not at pH 6.0 (10 ± 4%, *p* > 0.05).

### Effects of ranolazine on slow inactivation

**Figure 8:**
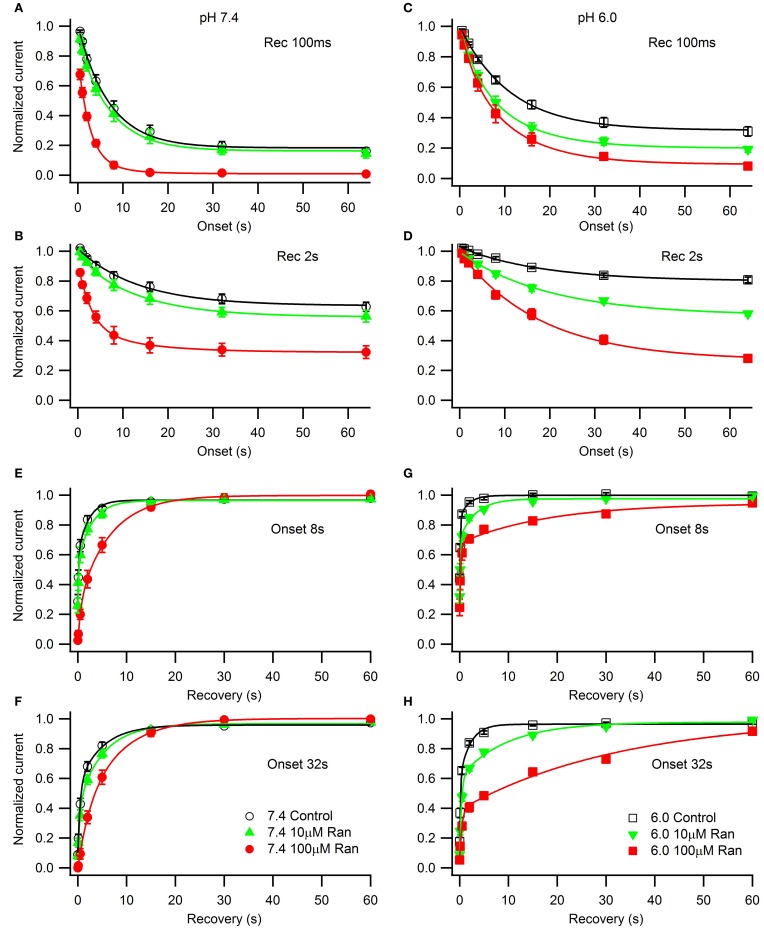
**Slow inactivation onset and recovery and effects of ranolazine at pH 7.4 (left) and 6.0 (right)**. Onset of slow inactivation **(A–D)** was induced by conditioning pulses to −10 mV ranging from 500 ms to 64 s. Amount of slow-inactivated channels (control, open black symbols) or a combination of slow-inactivated and ranolazine blocked channels (closed symbols) was assessed by 5 ms test pulses to −10 mV after either 100 ms **(A,C)** or 2 s **(B,D)** recovery at −130 mV. Onset kinetics were fitted with a single exponential function for control conditions and with a double exponential function for ranolazine conditions. Fit parameters are summarized in Table A7. Slow inactivation and ranolazine block recovery **(E–H)** was measured with a series of 5 ms test pulses ranged from 20 ms to 60 s after either 8 s **(E,G)** or 32 s **(F,H)** onset. Recovery followed double exponential kinetics in all conditions. Fit parameters are summarized in Table A8.

We studied the effects of acidic pH on ranolazine modulation of slow inactivation. The kinetics of onset and recovery from slow inactivation states and ranolazine block were assessed using a triple pulse protocol. Cells were given conditioning pulses to −10 mV varying in length from 500 ms to 64 s, followed by a −130 mV recovery pulse. Available current was measured with 5 ms test pulses to −10 mV applied at 20, 100, 500 ms, 2, 5, 15, 30 and 60 s after the end of the conditioning pulse (Figure 8). Here we report data from the 100 ms and 2 s recovery intervals (Figures 8A–D). These recovery periods allowed us to concurrently assess the slow inactivation onset and the onset of ranolazine block.

In drug-free conditions, slow inactivation onset followed a single exponential time course that was faster at pH 7.4 (τ_1_ = 7.0 ± 0.5 s, Figure 8A, open circles) than at pH 6.0 (τ_1_ = 11.2 ± 0.5 s, Figure 8C, open squares). Ranolazine causes an additional decrease in test pulse current amplitudes (Figures 8A–D, solid symbols). This reduction of channel availability in the presence of the drug was fitted with a double exponential function (Table A7). The onset of ranolazine block can be measured best with the 2 s recovery pulse. This time interval allowed near complete recovery from native slow inactivation but not from ranolazine block (Figures 8B,D). The drug-induced time constant of onset was significantly faster at pH 7.4 (100 μM: 3.0 ± 0.3 s, Figure 8B, solid circles) than at pH 6.0 (100 μM: 18 ± 2 s, Figure 8D, solid squares). We also observed a statistically significant effect of 10 μM ranolazine at pH 6.0 (Figures 8C,D; Table A7).

Recovery from slow inactivation and ranolazine block was observed after 8 or 32 s conditioning pulses (Figures 8E–H). Recovery followed double exponential kinetics in all conditions (Table A8). In drug-free conditions, recovery was faster at low pH. A distinct ultra slow recovery component developed in the presence of ranolazine. Interestingly, this drug-induced component of recovery was 4–5 fold slower at pH 6.0 than at pH 7.4. Thus, acidic pH not only impairs native Na_v_1.5 slow inactivation but also affects the interaction between the channel and ranolazine, making both onset and recovery of the drug effect considerably slower (Figure 8, Tables A7, A8).

**Figure 9:**
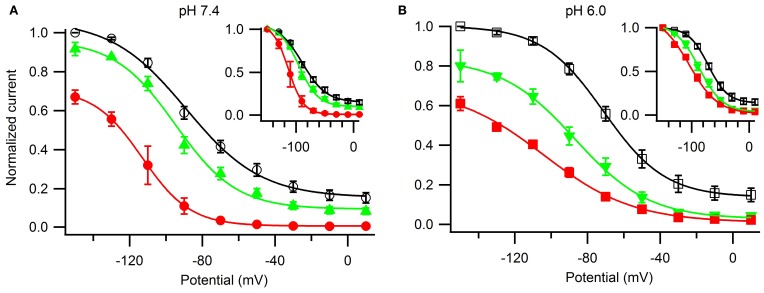
**The effects of ranolazine on steady-state slow inactivation and tonic block**. Slow inactivation was induced with alternating 30 s conditioning pulses to a range of voltages (−150 mV through +10 mV in 10 mV intervals) followed by a 20 ms recovery period at −130 mV to allow recovery from fast inactivation, and a −10 mV test pulse to measure channel availability. Channels spent additional 30 s at holding potential −130 mV between the sweeps. Matched pair experiments before and after ranolazine perfusion were performed to assess tonic inhibition by the drug at −150 mV (pH 7.4: 10 μM 8 ± 3%, *n* = 3, *p* = 0.001; 100 μM 33 ± 4%, *n* = 4, *p* < 0.001; pH 6.0: 10 μM 20 ± 8%, *n* = 3, *p* = 0.01; 100 μM 39 ± 4%, *n* = 6, *p* < 0.001). Currents were normalized to the maximum current in the absence of ranolazine **(A,B)** or to the maximum value at −150 mV (insets) and plotted as a function of the conditioning potential for control (open black symbols), 10 μM ranolazine (solid green triangles), and 100 μM ranolazine (solid red symbols). Data were fitted with a Boltzmann equation. Fit parameters are listed in Table A9. The differences between control and 10 μM ranolazine at pH 7.4 were not statistically significant (*p* > 0.05).

Lastly, we examined the voltage-dependence of steady-state slow inactivation and its modulation by ranolazine (Figure 9). Cells were given a 30 s conditioning pulse to potentials ranging from −150 to +10 mV in 10 mV intervals, then stepped to −130 mV for 20 ms to recover fast-inactivated channels, and finally a test pulse to −10 mV. Channels were allowed to recover for 30 s at −130 mV between the sweeps. Experiments immediately before and after drug perfusion were performed in a limited number of cells to estimate the amount of tonic inhibition by ranolazine at −150 mV (pH 7.4: 10 μM 8 ± 3%, *n* = 3, 100 μM 33 ± 4%, *n* = 4; pH 6.0: 10 μM 20 ± 8%, *n* = 3, 100 μM 39 ± 4%, *n* = 6). pH did not significantly affect tonic block by ranolazine. Steady-state availability curves normalized to peak availability in the absence of ranolazine are plotted in Figure 9. The insets show curves normalized to the respective current maxima at −150 mV.

In drug-free conditions acidic pH depolarized the steady-state availability by 18 mV (Table A9). 100 μM ranolazine produced dramatic leftward shifts at both pH 7.4 and 6.0 and eliminated the plateaus of the availability curves at depolarized potentials (Figure 9). 10 μM ranolazine at acidic pH significantly left shifted the availability curve by ~16 mV.

## Discussion

We examined the effects of ranolazine on Na_v_1.5 channels transiently expressed in HEK 293 cells at pH 7.4 and 6.0. We found that the kinetics of drug-channel interaction depends on pH conditions. Both the onset of and recovery from ranolazine modulation are slowed by 4–5 fold at pH 6.0 relative to pH 7.4 (Figures 4, 8). Ranolazine significantly reduced late/window sodium current at both pH conditions (Figures 6, 7).

### Physiological effects of extracellular pH

Extracellular pH plays a significant role in controlling activity of many physiological processes. Normal extracellular pH is ~7.4. Pathological conditions, such as hypoxia and/or ischemia decrease extracellular pH. Acidification decreases peak conductance of Na_v_ channels by protonation of outer vestibule carboxylates (Khan et al., 2002, 2006; Vilin et al., 2012) and also causes a depolarizing shift in the voltage-dependence of gating by surface charge screening (Hille, 1968; Benitah et al., 1997). In Na_v_1.5, acidosis increases persistent I_Na_ (Figure 6), which is considered to be a predisposing factor for cardiac arrhythmias (Amin et al., 2010; Jones et al., 2011). Low extracellular pH affects not only sodium channels. Peak current is reduced and kinetic changes are induced by low extracellular pH in a wide range of potassium channels (Deutsch and Lee, 1989; Kehl et al., 2002; Trapani and Korn, 2003). Similarly, calcium channels and the sodium/calcium exchanger (NCX) are affected by low extracellular pH in ways that are both direct and, in the case of NCX, at least partly related to changes in sodium channel behavior (see Carmeliet, 1999, for review). Our experimental system isolates sodium channels and thus provides only one facet of a complex suite of effects induced by low extracellular pH.

### pH effects on use-dependence in ranolazine

It is well-established that ranolazine at high concentrations (such as 100 μM) causes prominent use-dependent inhibition in various types of sodium channels at physiological extracellular pH (Fredj et al., 2006; Rajamani et al., 2008, 2009; Kahlig et al., 2010; Huang et al., 2011; Hirakawa et al., 2012). Consistent with these previous reports, we found that at extracellular pH 7.4 100 μM ranolazine produced substantial use-dependent block at 10 or 25 Hz (Figures 4A,B). At extracellular pH 6.0 use-dependent block with 100 μM ranolazine is substantially diminished at both frequencies (Figures 4C,D). The kinetics of use-dependence is also greatly slowed at acidic pH (Figure 4, Table A5).

The loss of channel availability during the pulse trains occur due to onset of ranolazine inhibition during the depolarizing pulses, and to slow recovery of channels from ranolazine-induced block between the pulses. In the presence of 100 μM ranolazine at pH 7.4 a pronounced slow component of recovery after 500 ms conditioning is evident (Figure 3A). Such a distinct slow component cannot be observed at pH 6.0 (Figure 3B). We hypothesized that its absence is due to slow kinetics of onset at acidic pH: a 500 ms conditioning pulse is insufficient to cause a detectable degree of inhibition by 100 μM ranolazine.

Prolonged trains of long (300 ms) and low frequency (1 Hz) depolarizations approximating cardiac action potential pace and duration demonstrate that 100 μM ranolazine produces substantial inhibition of sodium current even at pH 6.0 (Figure 5). This inhibition develops at a rate that is ~5 fold slower at pH 6.0 than at pH 7.4 but reaches a comparable magnitude after 500 s. The slow kinetics of onset at pH 6.0 explains the observed lack of effects of 100 μM ranolazine in short pulse duration protocols (Figures 1–3) and the reduced affects on high frequency use-dependence (Figure 4).

However, the effects of ranolazine on use-dependent reduction of peak sodium current at concentrations as high as 100 μM might not be therapeutically relevant. Ranolazine undergoes extensive biotransformation, primarily via CYP3A-mediated pathways of metabolism (Chaitman, 2006). Less than 7% of the parent compound remain un-metabolized (Jerling and Abdallah, 2005). Thus, with now commonly used sustained-release ranolazine, the concentration in patients' blood serum reaches only 2–6 μM (Chaitman, 2006; Antzelevitch et al., 2011). In our experimental conditions a therapeutically relevant concentration (10 μM) did not produce a significant use-dependent block of peak I_Na_ at either pH (Figures 4, 5). This finding might be important in the context of cardiac therapy since a prominent use-dependent block in the ischemic condition can be especially pro-arrhythmic (Moreno et al., 2011). The action of ranolazine in acidic conditions suggests the drug as ever more promising in the setting of ischemia due to stabilization of the inactivation state and, critically important, a reduction in the extent of use-dependent block.

### Impairment of inactivation states by acidic pH and its restoration by ranolazine

Acidic pH 6.0 impairs Na_v_ channel inactivation states causing depolarizing shifts in the steady-state profiles, slowing the onset of, and accelerating the recovery from both fast and slow inactivation. Addition of ranolazine selectively offsets the effects of low pH on slow inactivation. Therapeutically relevant ranolazine concentration (10 μM) at pH 6.0 essentially negates the effects of acidic pH (positive 18 mV shift, Figures 9A,B, open symbols) on the steady-state availability curve (pH 7.4: control V_1/2_ = −89 ± 3 mV; pH 6.0: 10 μM ranolazine V_1/2_ = −87 ± 2 mV). Although the onset and recovery drug kinetics are 4–5 fold slower at pH 6.0 (Figure 8), the *K*_d_-value at steady-state is expected to be in the similar range in both pH conditions.

In situations where Na_v_ fast inactivation is impaired and unable to shut off the ion flux through the pore slow inactivation can act as a “fail safe mechanism.” Consistent with this view, we suggest the stabilizing effect of ranolazine on slow inactivation reduces channel availability in a voltage-dependent manner (Figure 9) leading to a decrease in persistent (Figure 6) and window (Figure 7) sodium currents at both pH conditions.

### On the mechanism of action of ranolazine

Ranolazine action on Na_v_ channels is commonly viewed within the framework of the Modulated Receptor hypothesis (Hille, 1977), and presumed to be similar to local anesthetic drugs that were extensively studied over the past 3 decades (see Fozzard et al., 2005, for review). According to this paradigm, drug affinity is dependent upon the conformational state of the channel. Resting channels display lower affinity while open or inactivated channels have higher drug affinity. Within this framework, ranolazine use-dependent inhibition is often attributed to open channel block (Rajamani et al., 2008; Wang et al., 2008; Huang et al., 2011). This interpretation also assumes that binding of the drug molecule to the channel physically obstructs (“blocks”) ion flow through the channel pore. While this presumption is not an essential element of Modulated Receptor hypothesis and has not been unequivocally proven, it is often taken for granted. An alternative concept, “gating modifier,” suggests that binding of a drug molecule does not block the ion flow through the pore *per se*, but modulates conformational balance of the channel's intrinsic states. Most of the agents recognized as gating modifiers at present are toxins (Swartz and MacKinnon, 1997; Sokolov et al., 2008; Wang et al., 2011; Zhang et al., 2011) that interact primarily with voltage-sensing domains and modulate activation or fast inactivation gating. Ranolazine, along with lacosamide (Errington et al., 2008), may represent a distinct class of gating modifiers that affect slow inactivation gating of the channel. A crystal structure of the channel drug complex could provide strong evidence in favor of either the blocker or gating modifier hypotheses; however, such a structure is not available at this time.

Our data suggest that ranolazine does not block open Na_v_ channels. Instead, we propose that ranolazine induces a slow inactivation-like state with slow onset kinetics (seconds at pH 7.4, tens of seconds at pH 6.0), slow recovery kinetics (tens of seconds), and sharing the voltage-dependent profile of Na_v_ channel's intrinsic slow inactivation. First, our steady-state slow inactivation protocol featuring 30 s conditioning pulses demonstrates a significant reduction of channel availability at negative voltages where channel openings do not occur or are extremely rare (Figure 9). Second, in our matched ramp experiments, we observed a significant reduction of inward sodium currents during 500 ms ramp depolarizations (Figure 7). This result suggests the drug is already bound at voltages as negative as −130 mV rather than binding to open/inactivating channels during the ramp. Hence, 500 ms is a relatively short time for development of ranolazine block as revealed by the slow onset kinetics described in Figure 8.

Additional arguments for a specific interaction between ranolazine and slow inactivation come from previous studies. Multiple mutations in various Na_v_ channels that show increased persistent current also demonstrated increased sensitivity to ranolazine (Na_v_1.5 LQT3: ΔKPQ Fredj et al., 2006; Y1767C Huang et al., 2011; R1623Q Rajamani et al., 2009; Na_v_1.1 GEFS^+^, SMEI, FHM3: Kahlig et al., 2010). These mutations typically destabilize Na_v_ channel fast inactivation but ranolazine's effectiveness may be owing to its property to boost the “fail safe” slow inactivation mechanism. Recent studies of El-Bizri et al. (2011) in Na_v_1.4 and Hirakawa et al. (2012) in Na_v_1.3 demonstrated that ranolazine's effect on steady-state channel availability progressively increases with longer conditioning pulses, consistent with selective interaction between ranolazine and the slow-inactivated states.

Our results suggest that hNa_v_1.5/β1 heterologously expressed in HEK293 cells are affected by ranolazine in a manner similar to ventricular sodium channels as opposed to sodium channels in atria. In canine atrial myocytes, Burashnikov and Antzelevitch (2008) demonstrated substantial use-dependent block by 10 μM ranolazine as well as fast onset kinetics. In our experimental setup, the lack of significant use-dependent block at 10 μM and slow kinetics of drug-channel interaction suggests that atrial sodium channels differ substantially from the heterologously expressed hNa_v_1.5/β 1. Such difference may be attributed to tissue-specific cardiac sodium channel isoforms or differences in the stoichiometry of auxiliary subunits (Burashnikov and Antzelevitch, 2008, 2009, 2010).

Our study was limited to the effects of ranolazine on extracellular changes in pH. Ischemia results *a priori* in intracellular pH changes. Although intracellular protons are extruded by the sodium/hydrogen exchanger, NHE1, low intracellular pH also affects myocardial contractility and may alter the biophysical properties of ion channels, including Na_v_1.5 (Clanachan, 2006). Nevertheless, intracellular proton buffering mechanisms may limit the effects of low pH_i_, relative to those of extracellular protons, on ion channel function, and extracellular pH has been shown to change to a greater extent than intracellular pH (Crampin et al., 2006).

In conclusion, our results demonstrate that ranolazine induces a slow inactivation-like state in hNa_v_1.5/β 1 leading to reduction of late and window Na^+^ currents. The drug is especially effective at acidic extracellular pH where native slow inactivation is impaired. At pH 6.0 significant effects of the drug on kinetics and voltage-dependence of the drug-induced state can be observed at therapeutically relevant concentrations (10 μM), whereas open state and fast inactivation are not appreciably affected.

### Conflict of interest statement

S. Rajamani is an employee of Gilead Sciences, Inc, the producer of Ranolazine. This work was funded in part by a grant from Gilead Sciences, Inc. to P.C. Ruben. The other authors declare that the research was conducted in the absence of any commercial or financial relationships that could be construed as a potential conflict of interest.

## Appendix

**Table A1:** **Conductance**.

	**pH 7.4 Control**	**pH 7.4 (10 μM) Ranolazine**	**pH 7.4 (100 μM) Ranolazine**	**pH 6.0 Control**	**pH 6.0 (10 μM) Ranolazine**	**pH 6.0 (100 μM) Ranolazine**
V_1/2_	−44.6 ± 0.4^#^	−41.9 ± 0.4^*^	−40.9 ± 0.3^*^	−37.2 ± 0.3^*^^#^	−38.5 ± 0.3	−36.9 ± 0.3
z	8.4 ± 0.4	8.0 ± 0.4	7.4 ± 0.3	7.1 ± 0.3	7.1 ± 0.2	7.3 ± 0.3
*n*	13	4	6	20	8	13

* *Statistically different from control (p < 0.05)*.

# *Statistically different between non-drug pH conditions (p < 0.05)*.

**Table A2:** **Time constants of fast inactivation**.

	**pH 7.4 Control**	**pH 7.4 (100 μM) Ranolazine**	**pH 6.0 Control**	**pH 6.0 (100 μM) Ranolazine**
τ_−20 mV_	0.82 ± 0.04^#^	0.89 ± 0.06	1.52 ± 0.07^#^	1.73 ± 0.12
τ_−10 mV_	0.57 ± 0.03^#^	0.65 ± 0.05	1.05 ± 0.06^#^	1.11 ± 0.06
τ_0 mV_	0.50 ± 0.03^#^	0.47 ± 0.04	0.77 ± 0.03^#^	0.79 ± 0.05
τ_+10 mV_	0.40 ± 0.03^#^	0.39 ± 0.03	0.61 ± 0.02^#^	0.57 ± 0.02
*N*	13	9	21	10

# *Statistically different between pH conditions (p < 0.05)*.

**Table A3:** **Steady state fast inactivation**.

	**pH 7.4 Control**	**pH 7.4 (100 μM) Ranolazine**	**pH 6.0 Control**	**pH 6.0 (100 μM) Ranolazine**
V_1/2_	−96.8 ± 0.3^#^	−96.8 ± 0.5	−86.9 ± 0.2^#^	−84.8 ± 0.2
*z*	7.9 ± 0.3	7.9 ± 0.4	7.6 ± 0.2	7.4 ± 0.3
*n*	16	10	8	9

# *Statistically different between pH conditions (p < 0.05)*.

**Table A4:** **Recovery from fast inactivation**.

	**pH 7.4 Control**	**pH 7.4 (10 μM) Ranolazine**	**pH 7.4 (100 μM) Ranolazine**	**pH 6.0 Control**	**pH 6.0 (10 μM) Ranolazine**	**pH 6.0 (100 μM) Ranolazine**
τ_1_, ms	8.7 ± 0.9^#^	8.4 ± 0.7^#^	8.3 ± 0.5^#^	4.0 ± 0.4^#^	5.4 ± 0.5^#^	3.4 ± 0.03^#^
A_1_	0.61 ± 0.03	0.76 ± 0.03	0.51 ± 0.01	0.57 ± 0.02	0.71 ± 0.02	0.62 ± 0.02
τ_2_, ms	130 ± 20	200 ± 60	420 ± 50^*^	120 ± 20	140 ± 30	110 ± 20
A_2_	0.35 ± 0.03	0.24 ± 0.03	0.49 ± 0.02^*^	0.41 ± 0.02	0.27 ± 0.02	0.37 ± 0.02
*n*	4	4	9	6	5	8

* *Statistically different from control (p < 0.05)*.

# *Statistically different between pH conditions (p < 0.05)*.

**Table A5:** **Use dependence 10 and 25 Hz**.

	**pH 7.4 Control**	**pH 7.4 (10 μM) Ranolazine**	**pH 7.4 (100 μM) Ranolazine**	**pH 6.0 Control**	**pH 6.0 (10 μM) Ranolazine**	**pH 6.0 (100 μM) Ranolazine**
10 Hz, τ_1_, pulses			13.6 ± 0.2^#^			64 ± 3^#^
10 Hz, 100th pulse	0.91 ± 0.03	0.85 ± 0.03	0.33 ± 0.01^*^^#^	0.90 ± 0.02	0.89 ± 0.01	0.70 ± 0.01^*^^#^
10 Hz, *n*	5	6	6	7	6	9
25 Hz, τ_2_, ms			15.3 ± 0.2^#^			67 ± 4^#^
25 Hz, 100th pulse	0.85 ± 0.02	0.79 ± 0.04	0.26 ± 0.03^*^^#^	0.90 ± 0.02	0.90 ± 0.01	0.71 ± 0.01^*^^#^
25 Hz, *n*	6	5	6	6	6	6

* *Statistically different from control (p < 0.05)*.

# *Statistically different between pH conditions (p < 0.05)*.

**Table A6:** **Use-dependence 1 Hz**.

	**pH 7.4 Control**	**pH 7.4 (10 μM) Ranolazine**	**pH 7.4 (100 μM) Ranolazine**	**pH 6.0 Control**	**pH 6.0 (10 μM) Ranolazine**	**pH 6.0 (100 μM) Ranolazine**
τ_1_, pulses			4.3 ± 0.1^#^			25 ± 2^#^
τ_2_, pulses			530 ± 40^#^			320 ± 20^#^
500th pulse	0.82 ± 0.02	0.86 ± 0.03	0.49 ± 0.04^*^	0.84 ± 0.03	0.79 ± 0.03	0.50 ± 0.04^*^
*n*	7	6	9	3	7	4

* *Statistically different from control (p < 0.05)*.

# *Statistically different between pH conditions (p<0.05)*.

**Table A7:** **Slow inactivation onset**.

	**pH 7.4 Control**	**pH 7.4 (10 μM) Ranolazine**	**pH 7.4 (100 μM) Ranolazine**	**pH 6.0 Control**	**pH 6.0 (10 μM) Ranolazine**	**pH 6.0 (100 μM) Ranolazine**
Rec 100 ms, τ_1_, s	7.0 ± 0.5^#^	7.0^#^	7.0^#^	11.2 ± 0.5^#^	11.2^#^	11.2^#^
Rec 100 ms, A_1_	0.76 ± 0.02	0.75 ± 0.03	0.09 ± 0.03	0.65 ± 0.01	0.53 ± 0.07	0.63 ± 0.07
Rec 100 ms, τ_2_, s	N/A	0.61 ± 0.71	2.5 ± 0.2	N/A	3.9 ± 1.1	3.2 ± 1.0
Rec 100 ms, A_2_	N/A	0.11 ± 0.09	0.57 ± 0.03	N/A	0.22 ± 0.06	0.22 ± 0.06
Rec 100 ms, asymptote	0.18 ± 0.2	0.16 ± 0.1	0.01 ± 0.01^*^^#^	0.32 ± 0.01	0.20 ± 0.01^*^	0.09 ± 0.01^*^^#^
Rec 2s, τ_1_, s	13.4 ± 1.5^#^	13.4^#^	13.4^#^	18 ± 1^#^	18^#^	18^#^
Rec 2s, A_1_	0.37 ± 0.01	0.39 ± 0.01	0.15 ± 0.03	0.22 ± 0.01	0.22 ± 0.01	0.20 ± 0.01
Rec 2s, τ_2_, s	N/A	1.8 ± 0.7	3.0 ± 0.3^#^	N/A	22 ± 4	18 ± 2^#^
Rec 2s, A_2_	N/A	0.07 ± 0.01	0.45 ± 0.02	N/A	0.20 ± 0.01	0.50 ± 0.02
Rec 2s, asymptote	0.63 ± 0.01	0.56 ± 0.01	0.32 ± 0.01^*^	0.80 ± 0.01	0.57 ± 0.01^*^	0.27 ± 0.02^*^
*n*	6	9	4	6	7	5

* *Statistically different from control (p < 0.05)*.

# *Statistically different between pH conditions (p < 0.05)*.

**Table A8:** **Slow inactivation recovery**.

	**pH 7.4 Control**	**pH 7.4 (10 μM) Ranolazine**	**pH 7.4 (100 μM) Ranolazine**	**pH 6.0 Control**	**pH 6.0 (10 μM) Ranolazine**	**pH 6.0 (100 μM) Ranolazine**
Onset 8s, τ_1_, s	0.13 ± 0.03	0.12 ± 0.03	0.7 ± 0.2^*^	0.12 ± 0.01	0.14 ± 0.03	0.20 ± 0.07
Onset 8s, A_1_	0.31 ± 0.04	0.29 ± 0.03	0.23 ± 0.04	0.40 ± 0.02	0.45 ± 0.04	0.42 ± 0.04
Onset 8s, τ_2_, s	2.1 ± 0.4	2.9 ± 0.4	6.5 ± 0.5^*^^#^	1.8 ± 0.5	3.3 ± 0.9	18 ± 9^*^^#^
Onset 8s, A_2_	0.37 ± 0.04	0.42 ± 0.03	0.73 ± 0.04	0.15 ± 0.02	0.27 ± 0.04	0.27 ± 0.05
Onset 32s, τ_1_, s	0.45 ± 0.09^#^	0.4 ± 0.1	2.3 ± 0.5^*^	0.15 ± 0.03^#^	0.37 ± 0.09	0.4 ± 0.1^*^^#^
Onset 32s, A_1_	0.33 ± 0.05	0.33 ± 0.05	0.30 ± 0.09	0.43 ± 0.05	0.50 ± 0.06	0.32 ± 0.04
Onset 32s, τ_2_, s	5 ± 1	5.2 ± 0.8	7.4 ± 0.6^#^	2.1 ± 0.04	9 ± 3	33 ± 9^*^^#^
Onset 32s, A_2_	0.41 ± 0.06	0.54 ± 0.05	0.70 ± 0.09	0.36 ± 0.04	0.37 ± 0.05	0.62 ± 0.09
Onset 32s, *n*	6	8	4	6	6	5

* *Statistically different from control (p < 0.05)*.

# *Statistically different between pH conditions (p < 0.05)*.

**Table A9:** **Steady state slow inactivation**.

	**pH 7.4 Control**	**pH 7.4 (10 μM) Ranolazine**	**pH 7.4 (100 μM) Ranolazine**	**pH 6.0 Control**	**pH 6.0 (10 μM) Ranolazine**	**pH 6.0 (100 μM) Ranolazine**
V_1/2_	−89 ± 3^#^	−95 ± 3	−113 ± 1^*^^#^	−71 ± 1^#^	−87 ± 2^*^	−104 ± 4^*^^#^
z	21 ± 3	17 ± 2	13.3 ± 0.2^*^^#^	16.6 ± 0.4	20 ± 2	25 ± 3^*^^#^
*n*	13	9	7	13	9	8

* *Statistically different from control (p < 0.05)*.

# *Statistically different between pH conditions (p < 0.05)*.
